# Observation of intervalley quantum interference in epitaxial monolayer tungsten diselenide

**DOI:** 10.1038/ncomms9180

**Published:** 2015-09-01

**Authors:** Hongjun Liu, Jinglei Chen, Hongyi Yu, Fang Yang, Lu Jiao, Gui-Bin Liu, Wingking Ho, Chunlei Gao, Jinfeng Jia, Wang Yao, Maohai Xie

**Affiliations:** 1 Physics Department, The University of Hong Kong, Pokfulam Road, Hong Kong; 2 Center of Theoretical and Computational Physics, The University of Hong Kong, Pokfulam Road, Hong Kong; 3 Key Laboratory of Artificial Structures and Quantum Control (Ministry of Education), Department of Physics and Astronomy, Shanghai Jiao Tong University, 800 Dongchuan Road, Shanghai 200240, China; 4 School of Physics, Beijing Institute of Technology, Beijing 100081, China; 5 Innovation Center of Advanced Microstructures, Nanjing, China

## Abstract

The extraordinary electronic structures of monolayer transition metal dichalcogenides, such as the spin–valley-coupled band edges, have sparked great interest for potential spintronic and valleytronic applications based on these two-dimensional materials. In this work, we report the experimental observation of quasi-particle interference patterns in monolayer WSe_2_ using low-temperature scanning tunnelling spectroscopy. We observe intervalley quantum interference involving the Q valleys in the conduction band due to spin-conserving scattering processes, while spin-flipping intervalley scattering is absent. Our results establish unequivocally the presence of spin–valley coupling and affirm the large spin splitting at the Q valleys. Importantly, the inefficient spin-flipping scattering implies long valley and spin lifetime in monolayer WSe_2_, which is a key figure of merit for valley-spintronic applications.

## Abstract (web summary)

Monolayers of transition metal dichalcogenides have many of the properties required for applications in spintronics and valleytronics. Here, the authors experimentally observe intervalley quantum interference involving conduction band Q-valleys in tungsten diselenide.

## Graphical abstract

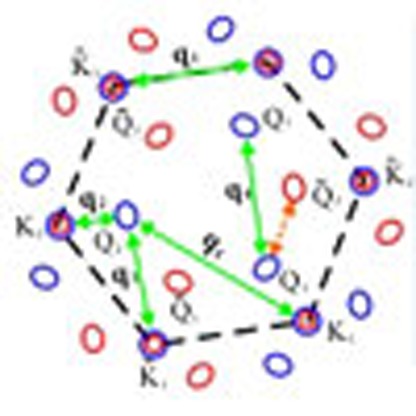

Monolayer (ML) transition metal dichalcogenides (TMDs) are attracting great research interests for their extraordinary properties, in particular the exotic spin–valley-coupled electronic structures that promise future spintronic and valleytronic applications^1^,^2^,^3^. The energy bands of ML TMDs have well-separated valleys that constitute effectively an extra internal degree of freedom for low-energy carriers. Besides the well-studied K valleys^3^,^4^,^5^,^6^,^7^,^8^,^9^,^10^,^11^,^12^, the conduction band also has six Q valleys located approximately midway between the Γ and K points of the first Brillouin zone (BZ), where interesting valley physics is also discovered^3^,^11^,^12^. The large spin–orbit coupling in the TMDs makes the spin index locked to the valley index for low-energy carriers, which has some interesting consequences such as the magnetoelectric effects in 2H bilayers^13^. Direct experimental characterizations of the spin–valley-coupled electronic structures are of great interests for both fundamental physics and device applications.

Scanning tunnelling microscopy/spectroscopy (STM/S) is a known powerful method for probing electronic structures of thin films and its studies have already been implemented to extract the quasi-particle band gaps and band edges in ML TMDs^14^,^15^,^16^,^17^,^18^. Quantum quasi-particle interference (QPI) has been studied by STM/S for metals^19^,^20^, topological insulators^21^,^22^ and graphene^23^. A defect elastically scatters electrons from wave vector **k**_i_ to **k**_f_ on the constant energy contour (CEC), and quantum interference of the incoming and scattered waves form standing waves of wave vector **q**=**k**_f_−**k**_i_ that are detected by low-temperature STS mapping. For multi-valley bands such as that in graphene and ML TMDs, the scattering by a point defect can give rise to rich QPI patterns arising from quantum interference within a valley as well as between well-separated inequivalent valleys. Fourier transform of the STS maps (FT-STS) can then directly reveal the valley locations and the band dispersion relations^24^,^25^, for example.

Here we report the experimental observation of the QPI pattern of electrons in ML WSe_2_ using a low-temperature STS. We observe intervalley quantum interference involving the Q valleys due to spin-conserving scattering processes, while spin-flipping intervalley scattering is absent. Our experiment establishes unequivocally the presence of spin–valley coupling and affirms the large spin splitting at the Q valleys. Importantly, the inefficient spin-flipping intervalley scattering implies long valley and spin lifetime in ML WSe_2_, which represents a key figure of merit for valley-spintronic applications.

## Results

### STM/S of MBE-grown WSe_2_

**Figure 1:**
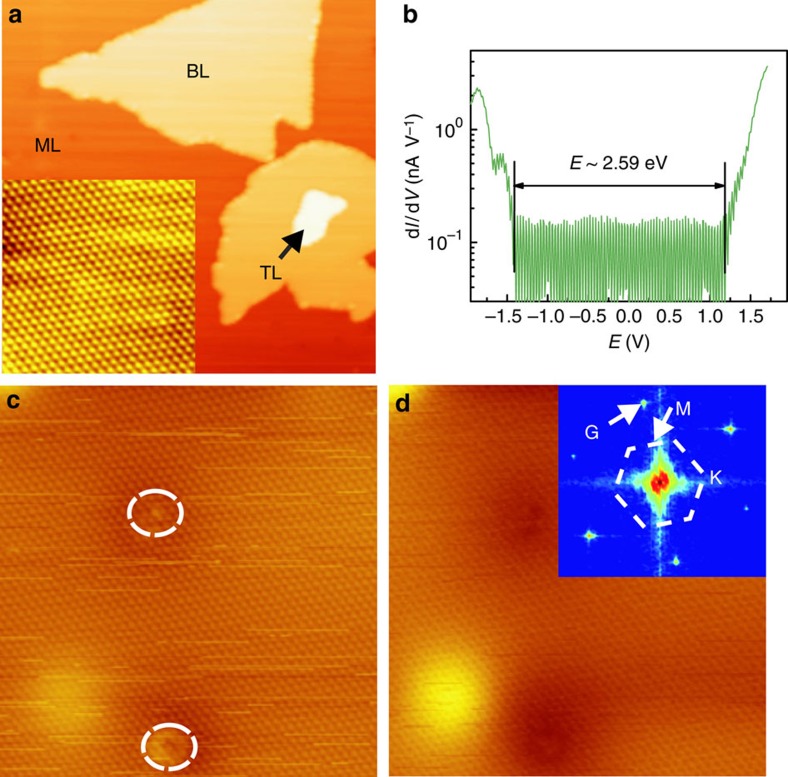
STM/S of MBE-grown WSe_2_. (**a**) STM topographic image (size: 75 × 75 nm^2^, sample bias: 2.4 V) of a MBE-grown WSe_2_ sample on highly ordered pyrolytic graphite with the nominal coverage of 1.2 MLs, revealing dominantly ML WSe_2_, bilayer (BL) and trilayer (TL) islands. Inset: an atomic resolution image (size: 7.5 × 7.5 nm^2^, sample bias: −1.5 V) of ML WSe_2_. (**b**) STS spectrum (averaged over 50 scans) taken at a fixed position on a defect-free ML WSe_2_, revealing an energy gap of 2.59 eV. (**c**) Topographic STM image and (**d**) STS map of ML WSe_2_ measured simultaneously at −1.5 eV (size: 14 × 14 nm^2^). The white dashed circles in (**c**) mark two point defects, which show darker contrast in (**d**). The inset in (**d**) is the FT-STS map showing the reciprocal lattice (the points labelled by G). The first Brillouin zone (the white dashed hexagon) and its high symmetry points (K and M) are also shown for reference.

Figure 1a shows a topographic image of an as-grown WSe_2_ sample. It reveals a characteristic terrace-and-step morphology of an atomically flat film (refer to Supplementary Figure 1 for the reflection high-energy electron diffraction (RHEED) patterns). The sample of Fig. 1a has the nominal coverage of 1.2 MLs, so in addition to the ML film over large surface areas, there are also bilayer and even trilayer high islands. Comparing with the molecular-beam epitaxy (MBE)-grown MoSe_2_ films^18^, the most striking feature in Fig. 1a is the absence of inversion domain boundary network commonly seen in epitaxial MoSe_2_. This has given us an opportunity to study the quantum interference effect in ultrathin WSe_2_ by STM/S, thereby allows probing the electronic structure as well as the spin splitting in ML TMDs. The inset in Fig. 1a is a close-up atomic resolution image of the ML region of the sample. Fig. 1b shows the differential conductance spectrum measured at a fix point on ML WSe_2_ by STS at 77 K. It reveals an energy gap of 2.59±0.07 eV, in agreement with the previous report^16^. The Fermi level is found slightly above the mid-gap energy, suggesting the sample is slightly electron doped, likely by some native defects such as Se vacancy.

### QPI in the valance band

To search for the QPI in ML WSe_2_, we first locate a surface area that contains point defects. An example is given in Fig. 1c, which shows two point defects in the field of view. In this region of the surface, quantum interference stands high chance to be observed by low-temperature STM/S. We began our search of the QPI by taking the STS maps at energies close to the valence band maximum (≤−1.4 eV). Figure 1d shows one of the STS maps obtained and the inset presents the power spectrum through Fourier transform. For all the STS maps taken in the energy range of −1.4 to −1.5 eV, including Fig. 1d, no sign of intervalley QPI is seen and the FT-STS only reveal the reciprocal lattice vectors (**G**) of WSe_2_. The latter has, nevertheless, allowed one to determine the Brillouin zone as marked by the white dashed hexagon in the figure.

**Figure 2:**
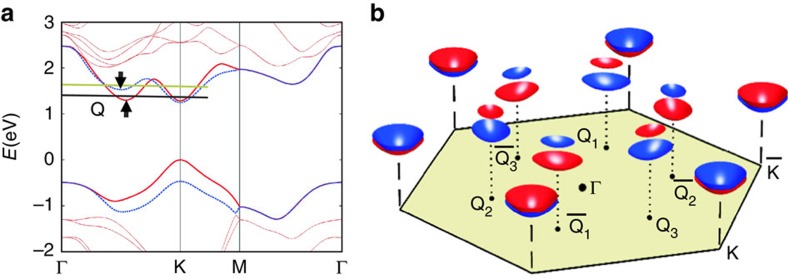
K and Q valleys in ML WSe_2_. (**a**) Electronic bands of ML WSe_2_ calculated by DFT. In the bottom conduction and top valence bands, the spin-down and -up subbands are denoted by red and blue colour, respectively. The vertical arrows point to the minima of the spin-split bands at Q valley. The two horizontal lines mark the energies at which the constant energy contour maps in Figs 3d and 4c are obtained. (**b**) Schematic illustration of the spin–valley-coupled conduction-band edges, where the blue and red colours denote the spin-up and spin-down bands, respectively.

The absence of intervalley QPI in the valence band implies that spin-flipping scattering between K and 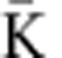 valleys is inefficient. In ML WSe_2_, the ultra-strong spin–orbit coupling in the 5d orbitals of the metal atoms give rise to large spin splitting in both the conduction and valence bands, which are dictated by the mirror symmetry and time-reversal symmetry to be in the opposite out-of-plane directions at a time-reversal pair of either K or Q valleys^3^. In Fig. 2a, we show electronic bands of ML WSe_2_ calculated by the density functional theory (DFT), in which the red solid and dotted blue lines represent the spin-split bands due to spin–orbit coupling. The valence band edge at the K points has a large spin splitting^17^,^26^ with opposite signs at K and 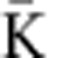 as required by the time-reversal symmetry^3^,^27^. Consequently, QPI between K and 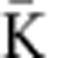 valleys will be prohibited by the time-reversal symmetry if the scattering defects are non-magnetic. Furthermore, a recent study has shown that STM/S is not very sensitive to the K-valley states as compared with the Γ and Q valleys^17^, which may be another cause for the absence of the QPI in STS near the valence band maximum.

### QPI of conduction-band electrons

**Figure 3:**
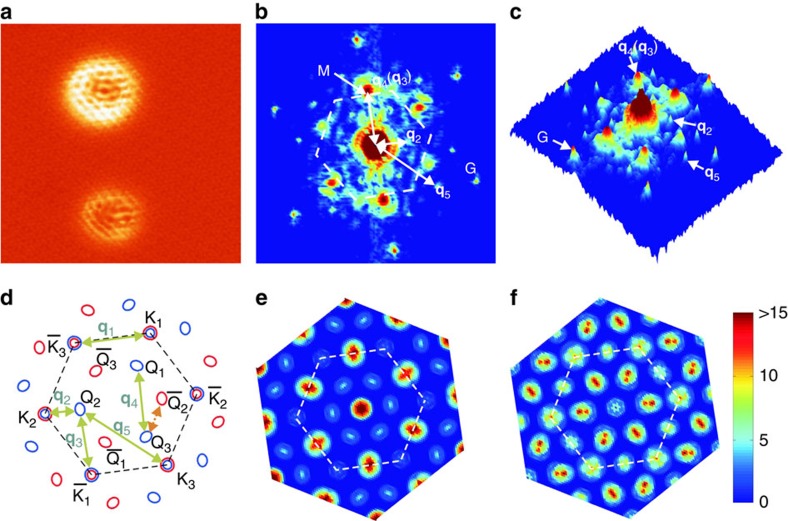
Intervalley quantum interference at defects. (**a**) STS map of ML WSe_2_ at +1.0 eV of the same surface as in Fig. 1c, showing QPI patterns in the vicinity of the two point defects. (**b**,**c**) The FT-STS map in (**a**) presented in the top and perspective views, respectively. The white dashed hexagon in (**b**) shows the first Brillouin zone . The observed scattering wave vectors (**q**_i_s) are exemplified by arrowed solid white lines in (**b**) and pointed in (**c**). The central ring-like feature as highlighted by the yellow dotted circle in (**b**) may reflect the intravalley scattering. (**d**) Constant energy contour at the energy marked by the black horizontal line in Fig. 2a. The blue (red) colour denotes the up (down) spin states. The solid green arrows indicate five possible spin-conserving scattering channels with the resulting interference wave vectors **q**_i_. The dashed orange arrow indicates a spin-flipping scattering channel. (**e**,**f**) Calculated joint density of states for, respectively, spin-conserving and spin-flipping scatterings at the same energy as in (**d**).

Conduction band electron has a completely different story. The spin splitting is much smaller at the K-point (∼0.03 eV), and the largest spin split of ∼0.2 eV occurs in the Q valley (cf. Fig. 2a). The energy minimum at Q is close to that at K for ML WSe_2_ (refs 3, 11, 17, 28, 29, 30, 31). Besides, Q valley can have a much larger weight in STM/S measurements than the K valley due to the larger density of states (DOS) as well as a larger tunnelling coefficient. Therefore, the Q valleys are expected to play a significant role in the STS mapping of the empty states. As illustrated in Fig. 2b, the six-fold degenerate Q valleys form two groups. Q_1_, Q_2_ and Q_3_ have the same spin and 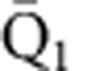, 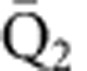 and 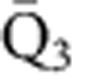 are their time reversals. QPIs by the spin-conserving intervalley scattering are possible within each group. In addition, as the spin splitting is small at K while the energy difference between Q and K is also small, QPIs by the spin-conserving scatterings between K and 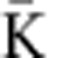, and between Q and K valleys may also occur (cf. Fig. 3d).

Figure 3a presents an example of the STS maps obtained over the same surface area as in Fig. 1c but at an energy of *E*=+1.0 eV. Unlike the filled-state STS map of Fig. 1d, the empty-state image reveals clearly the QPI patterns in the vicinity of the two defects. Figure 3b,c show the corresponding FT-STS maps presented in the top and perspective views, respectively, from which one notes distinct intensity spots besides the reciprocal lattice (**G**), signifying the various scattering channels and the corresponding standing wave vectors **q** as labelled. To identify the relevant scattering processes, we show in Fig. 3d a CEC map derived from the DFT calculations. This CEC map corresponds to an energy that is slightly above the conduction-band minimum (that is, the black horizontal line in Fig. 2a), where the spin texture of the Q valley is clearly resolved. For the K valley, on the other hand, both spin-up and spin-down bands are present with a small difference on the CEC. On the basis of this CEC, we may identify a number of possible spin-conserving intervalley scatterings as indicated by the green arrows. A spin-flipping inter-Q valley scattering is also marked by a dotted orange line. By comparing with the experimental data, we may assign the strongest scattering with the wave vector close to the M point of the Brillouin zone (that is, the middle point on the edge of the dashed white hexagon) is of the spin-conserving inter-Q valley scattering, that is, **q**_4_ in Fig. 3d.

A possible complication, however, is the presence of scattering between Q and K valleys, which would result in a wave vector (**q**_3_ in Fig. 3d) that is very similar to **q**_4_. With the resolution of the experimental data, we cannot discriminate one from the other, and indeed both could occur. Nevertheless, according to the band structure calculations^3^,^11^,^17^,^28^,^29^, the Q valley is situated slightly off the midpoint of Γ–K and closer to K (see Fig. 2a), so **q**_4_ would be slightly larger than 
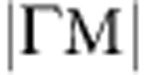
, which appears consistent with the experimental data. Moreover, as mentioned earlier, the STM/S measurement should be more sensitive to Q states than the K states, therefore the Q–Q QPI has a larger chance to be picked up in the STM/S images compared with the Q–K one. We therefore believe that Q–Q scattering 
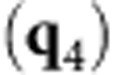
 is the dominating contributor to the experimentally observed spot next to M in Fig. 3b,c.

With the presence of both spin-up and -down subbands at K valleys and the six-fold degenerate Q valleys on the CEC, Q–K QPI at another two wave vectors, **q**_2_ and **q**_5_, and 
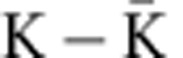
 QPI at wave vector **q**_1_ are also possible through the spin-conserving scattering (cf. Fig. 3d). Though weak, intensity spots are indeed observed at **q**_2_ and **q**_5_ as shown in Fig. 3b,c. Intensity spot at wave vector **q**_1_ may not be discernable in Fig. 3b or c, which may again be attributed to the sensitivity of STM/S to K-valley electrons. However, we did observe hint of such scatterings at some other energies (Supplementary Fig. 2).

### JDOS maps

**Figure 4:**
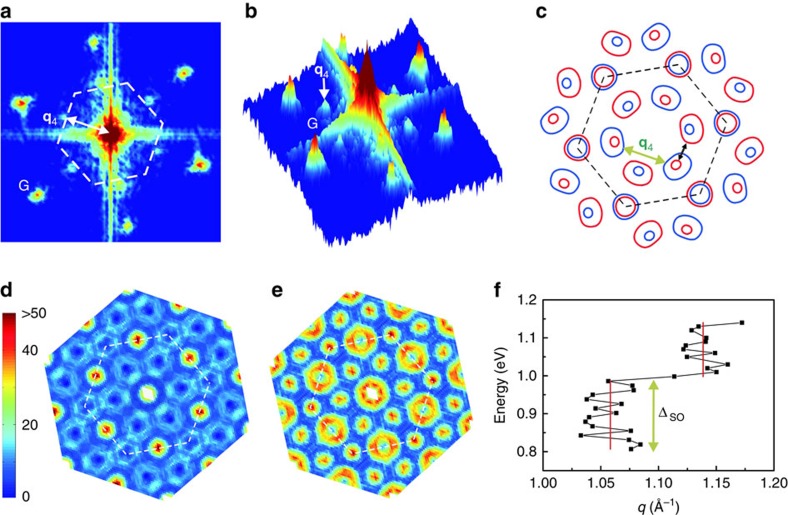
Energy dependence of the quantum interference pattern. (**a**,**b**) FT-STS map of the surface as in Fig. 3 but measured at energy +1.2 eV and presented in the top and perspective views, respectively. (**c**) Constant energy contour at an energy marked by the horizontal green line in Fig. 2a, where the blue (red) line denotes the up (down) spin states. The solid green arrow indicates a spin-conserving Q_i_−Q_j_ scattering channel. The black arrow indicates a spin-conserving 
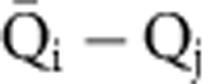
 intervalley scattering channel, as both spin-up and -down subbands at Q valleys are relevant at this higher energy. (**d**,**e**) The joint density of state maps calculated for, respectively, spin-conserving and spin-flipping scatterings at same the energy as in (**c**). (**f**) Experimentally derived wave vectors (**q**_4_) from the FT-STS maps at different energies (from 0.7 to 1.2 eV), revealing two branches as highlighted by the solid red lines. The vertical green arrow indicates the magnitude of spin splitting at Q.

We have performed calculations of the joint density of states (JDOS), 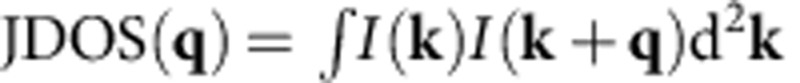, where *I*(**k**) represents the DOS derived from the DFT calculated energy bands. Figure 3e,f shows two JDOS maps at the energy of Fig. 3d but for spin-conserving and spin-flipping scatterings, respectively. For comparison and completeness, Fig. 4 presents another CEC map (4c) and the corresponding JDOS (4d and 4e for the spin-conserving and spin-flipping scatterings, respectively) for a higher energy (the green line in Fig. 2a) and compared with an experimental FT-STS obtained at 1.2 eV (4a and 4b for the top and perspective view, respectively).

## Discussion

Comparing the JDOS maps with experimental FT-STS, it is clear that the spin-conserving scattering captures well the experimental results, elucidating the non-magnetic nature of the defect. Should spin-flipping scattering processes be efficient, one would expect different FT-STS (that is, Figs 3f and 4e). Our experiment therefore affirms the spin-conserving scattering processes are much more efficient than the spin-flipping ones in ML WSe_2_. Together with the absence of inter-K valley scattering in the valence band as described earlier, it implies long valley lifetime, promising valleytronics applications based on valley polarization of the Q-valley electrons and K-valley holes.

The agreement between Fig. 3b,e also evidences the large spin splitting at Q in ML WSe_2_, as otherwise spin-conserving 
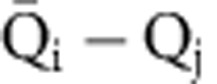
 scattering channels would be present over most of the energy range, contradicting to the experiment. By examining the STS maps obtained at different energies, we derive an energy dependence of the 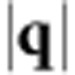
's (peak-to-peak distances in FT-STS maps). Figure 4f summarizes the data for the wave vector **q**_4_. There are apparently two branches, corresponding to 
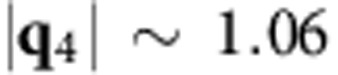
 Å^−1^ and 1.14 Å^−1^, respectively. According to the first-principle band structure, the exterma of the upper and lower spin subbands at the Q valley show a relative shift in momentum (as indicated by two short vertical arrows in Fig. 2a). Therefore, we are tempted to attribute the two branches in Fig. 4f to be scatterings dominantly in the lower and upper spin subbands, respectively. If this is indeed the case, we may further estimate the magnitude of the spin-split Δ_SO_ at Q, which is about 0.2 eV. This is an appreciable spin splitting as pointed out above, which compares well with the first-principle calculation estimations^11^. We, however, note a discrepancy between theory and the experiment, where most first-principle calculations suggest the upper spin subband at Q has a smaller momentum than the lower one, which is opposite to the finding of Fig. 4f. Besides, the experimental data can have contributions of the other scattering channels (for example, the Q–K scattering **q**_3_). Hence the two momentum branches observed in Fig. 4f may reflect a change of dominance of the different scattering processes when the energy is changed. Further studies by higher-resolution STM/S may resolve this ambiguity.

Finally, we would like to draw attention of the seemingly ring feature in the FT-STS map at the centre (see Fig. 3b,c). It may reflect intravalley scattering of electrons. Unfortunately, because of long wavelength (and thus small **q**), the ring feature cannot be separated well from the central bright spot often affected by the random noise of the STS maps. So we have not been able to derive the dispersion relation of the Q valley in this experiment.

It should also be noted that, during the review of this article, a study on exfoliated WSe_2_ with similar results for intervalley scattering was reported^32^.

## Methods

### Film growth

Ultrathin WSe_2_ films were grown on freshly cleaved highly ordered pyrolytic graphite in a customized Omicron MBE reactor with the base pressure of 10^−10^ mbar. Elemental Tungsten (W) of purity of 99.99% and Selenium (Se) of 99.999% in purity were used as the sources, and their fluxes were generated from an e-beam evaporator and a dual-filament Knudsen cell, respectively. A flux ratio of 1:15 between W and Se was used. The substrate temperature during film deposition was ∼400 °C and the deposition rate was 0.5 MLs per h. During growth, the film surface was monitored *in situ* by the RHEED operated at 10 KeV, which showed streaky patterns signifying two-dimensional layer-by-layer growth mode of WSe_2_ on highly ordered pyrolytic graphite. After a preset coverage of the deposit was grown, the source fluxes were cutoff by mechanical shutters. The film was subsequently annealed at ∼500 °C for 1 h before being brought to room temperature by natural cooling on shutting off the power to the sample manipulator.

### STM measurement

Room-temperature STM measurements were performed *in situ* using an Omicron VT-STM system while the low-temperature STM measurements were carried out at 77 K in a Unisoku low-temperature STM system *ex situ*. For the latter, the sample surface was protected by an amorphous Se layer deposited at room temperature until the RHEED pattern became featureless but a diffusive background. After transferred to the Unisoku low-temperature STM system, the sample was gently annealed at 300 °C until the streaky RHEED patterns reappeared and the same flat surface morphology recovered according to STM examinations. For both room-temperature and low-temperature STM measurements, the constant current mode was used. For STS measurements, the lock-in technique was employed using a modulation voltage of 15 mV and frequency of 985 Hz.

### DFT and JDOS calculations

The DFT calculations were done by the Vienna Ab-initio Simulation Package (VASP) code^33^ using the projector-augmented wave^34^ as well as the Perdew–Burke–Ernzerhof exchange-correlation functional^35^. Relaxed lattice parameters were used for WSe_2_ monolayer^28^. Spin–orbit coupling was considered in the calculation. The energy cutoff of the plane-wave basis was set to 400 eV and the energy convergence was 10^−6^ eV. Vacuum layer was >15 Å to separate neighbouring periodic images. A Γ-centred k-mesh of 10 × 10 × 1 was used to obtain the ground-state density and a 51 × 51 k-mesh was used to calculate the band energies in the rhombus reciprocal cell.

After getting the conduction-band dispersion 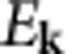 of the two-dimensional first Brillouin zone, the 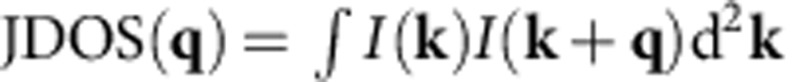 at the Fermi level *E*_F_ was calculated using the approximation 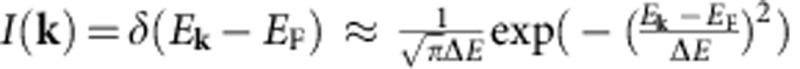. We set the energy resolution ▵*E*=30 meV, which suited well our 51 × 51 reciprocal cell mesh grid.

## Additional information

**How to cite this article:** Liu, H. *et al.* Observation of intervalley quantum interference in epitaxial monolayer tungsten diselenide. *Nat. Commun.* 6:8180 doi: 10.1038/ncomms9180 (2015).

## Supplementary Material

Supplementary Information Supplementary Figures 1-2
