# Supplementary material for: Observation of intervalley quantum interference in epitaxial monolayer tungsten diselenide
Source: Nat Commun. 2015 Sep 1;6:8180. doi: 10.1038/ncomms9180 (PMC4569794; doi:10.1038/ncomms9180)
Supplement: Supplementary Information — Supplementary Figures 1-2 [file ncomms9180-s1.pdf]

Supplementary Figures

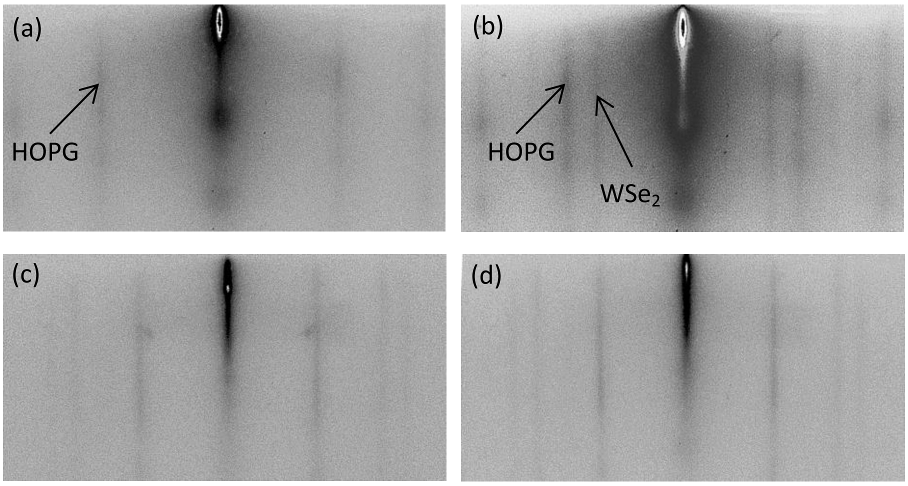

Supplementary Figure 1: **RHEED pattern evolution of WSe_2 growth** RHEED patterns were recorded at different deposition stages, from bare substrate to a film of 1.2 MLs, during MBE growth of WSe_2 on HOPG substrate. (a) is for a freshly treated HOPG substrate. (b) is for a surface after about 0.25 MLs WSe2 being deposited, where the diffraction streaks of HOPG and WSe_2 coexist. (c) is for the as-grown 1.2 ML WSe_2 sample, and (d) shows the pattern of the same sample as (c) after annealing. The streaky pattern indicates smooth surfaces and the layer-by-layer growth mode. Annealing of the grown sample further improves the surface morphology as indicated by sharpening of the RHEED streaks and affirmed by the STM measurements (refer to Fig. 1 of the main text)

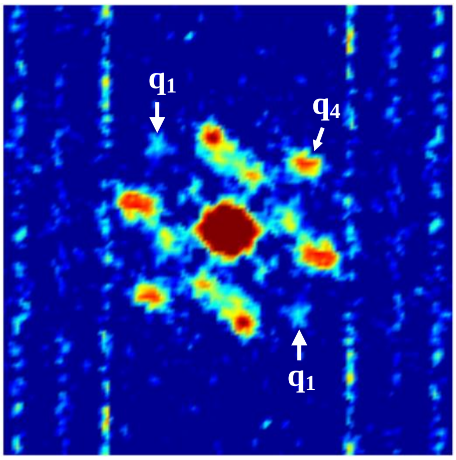

Supplementary Figure 2: **FT-STS map at 0.879 eV** Hint of inter-K valley scattering can be noted as labelled by $\mathbf{q_1}$. As in the main text, $\mathbf{q_4}$ labels the inter-Q valley scattering.
